# Trans‐omic analyses identified novel prognostic biomarkers for colorectal cancer survival

**DOI:** 10.1002/ctm2.1304

**Published:** 2023-06-16

**Authors:** Chanjuan Liu, Furong Yan, Dongli Song, Yiming Zeng, Tiankui Qiao, Duojiao Wu, Yunfeng Cheng, Hao Chen

**Affiliations:** ^1^ Center for Tumor Diagnosis & Therapy, Jinshan Hospital, Fudan University Shanghai China; ^2^ Department of Pulmonary and Critical Care Medicine, Zhongshan Hospital, Fudan University Shanghai China; ^3^ Center of Molecular Diagnosis and Therapy, The Second Hospital of Fujian Medical University Quanzhou Fujian China; ^4^ Shanghai Institute of Clinical Bioinformatics Shanghai China; ^5^ Department of Thoracic Surgery Zhongshan-Xuhui Hospital, Fudan University Shanghai China

Dear Editor,

Colorectal cancer (CRC) is third most frequently diagnosed malignancy with the second highest mortality rate among all cancers.^1^, ^2^, ^3^, ^4^ As CRC survival is highly dependent upon early diagnosis and treatment, novel sensitive and non‐invasive detective approaches f are urgently required.^5^ CRC is characterized by metabolic reprogramming.^6^, ^7^ In present study, using plasma metabolome analysis, a large scale trans‐omic network was constructed by connecting polar metabolite data, lipid data, clinical phenoms and survival outcomes of CRC patients. The study revealed that the excavation of trans‐omics based on serum metabolome could potentially predict CRC survival. We believe that this trans‐omics approach is vital for optimizing personalized treatment and the development of precision medicine for CRC.

The plasma samples were collected and analysed for metabolomic and lipidomic analysis. Our experiment consisted of two cohorts (Table S1). In cohort 1, 78 CRC patients and 78 healthy control (HC) samples were collected for lipidomics analysis. In cohort 2, samples from 31 CRC patients and 31 HC were collected for metabolomics analysis (Figure 1A). We found that the polar metabolites and lipids were significantly different between CRC patients and HC (Figure 1B–F). There were eight main classes of lipid detected in our study, including lysophosphatidylcholines, phosphatidylcholines (PC), phosphatidylethanolamine (PE), diacylglycerol, sphingomyelin (SM), cholesterol ester (CE), ceramide and triacylglyceride (TAG). The occurrence and prevalence of CRC are closely related to patient age.^8^ We further sub‐grouped the CRC patients by age into groups of adult (age less than or equal to 50 years) and senior (older than 50 years), and metabolic analysis was performed (Figure 1G). Volcano plots were generated by comparing the three groups in pairwise (Figure 1H). Multiple polar metabolites in the plasma were down‐regulated in both the CRC groups compared with the HC group (Figure 1I,K, L). The expression of 5‐aminovaleric acid and d‐allose in adult CRC patients was higher than that of HC and senior CRC patients (Figure 1J,L). Pathway enrichment analysis identified signalling pathways associated with key polar metabolites between groups (Figure 1M). Pathways of fatty acid degradation were enriched in both senior and adult CRC groups, compared with HC. The variable influence on projection (VIP) score can summarize the importance of each variable. Differentially expressed eight polar metabolites (VIP > 1, *p* < .05 and FC > 2 or <.5) had shown a great diagnostic potential for CRC (Figure 1N). Averaged ROC curves revealed that these polar metabolites had great diagnostic potential for CRC (Figure 1O). These findings strongly suggested the potential application of polar metabolites as clinical indicator for the early diagnosis of CRC.

**FIGURE 1:**
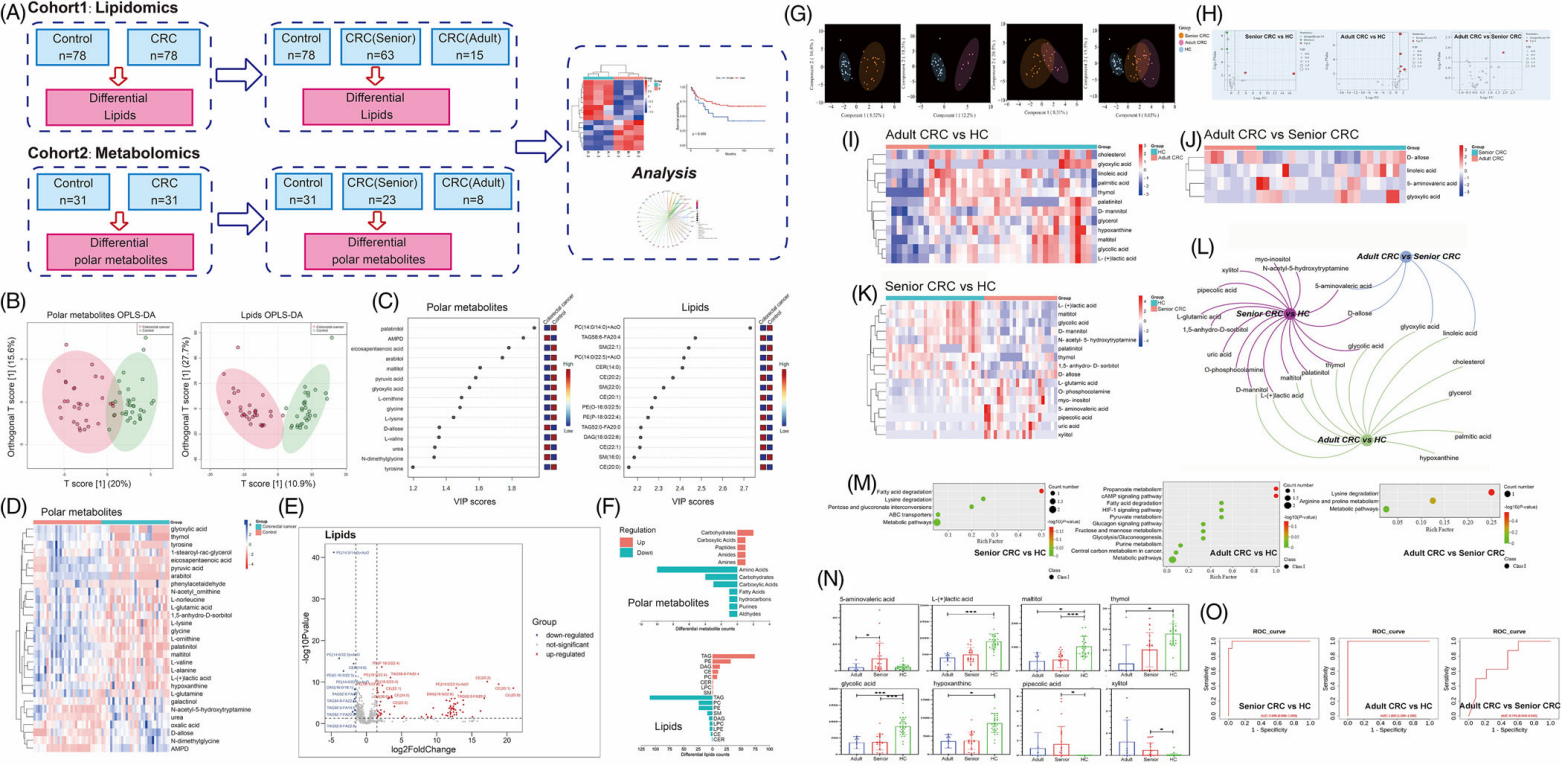
The metabolomic landscape of colorectal cancer (CRC). Blood samples from CRC patients and healthy control (HC) groups were collected and identified by LC–MS and GC–MS and used for trans‐omics analysis (A). Orthogonal partial least squares discriminant analysis (OPLS‐DA) score plot showed high separating capacity in lipidomics and metabolomics between CRC and HC group (B). The 15 top‐scoring variable influence on projection (VIP) polar metabolites and lipids were shown in bubble plots (C). Heat map showed the significantly up‐ /down‐regulated expressed polar metabolites in CRC group (*p* < .05, compared with HC, D). Volcano plot showed the significantly up‐ /down‐regulated expressed lipids in CRC group, comparing with HC group (E). Lipids and polar metabolites were categorized by their class. The total number of up‐ and down‐regulation of each class was also calculated (F). (G) CRC patients grouped by their ages as senior (older than 50 years) and adult (age less than or equal to 50 years). OPLS‐DA score plots were used for the pairwise difference polar metabolites analysis among adult CRC, senior CRC and HC groups. Adult CRC and control groups showed a distinct separation. The distinct separation of senior CRC and control groups also was observed. A separation of adult and senior CRC groups was also observed in OPLS‐DA score plot. (H)Volcano plots showed differences between pairwise comparison of the three groups (HC, adult CRC and senior CRC group). (I–K) Heat maps showed detailed differences among three groups (adult CRC vs. HC, adult CRC vs. senior CRC and senior CRC vs. HC, *p* < .05). (L) Bipartite graph of the differential polar metabolites from the pairwise comparison among three groups. The large node represents the contrasting groups, and the small node represents the differential polar metabolites. The connection between polar metabolites and contrasting groups suggested that the polar metabolite was the common differential metabolite. (M) The bubble plot showed the enrichment pathways of the significant polar metabolites between pairwise comparisons of the three groups. (N) The expression levels of these eight polar metabolites in control, adult CRC and senior CRC groups, respectively. Differential polar metabolites between the adult CRC and HC, senior CRC and HC, and adult CRC and senior CRC (VIP > 1, *p* < .05 and FC > 2 or <.5), a histogram was drew to illustrate the expression of metabolites (5‐aminovaleric acid, l‐(+)lactic acid, maltitol, thymol, glycolic acid, hypoxanthine, pipecolic acid and xylitol) in these three groups. (O) ROC analysis was applied to the metabolite data using the six polar metabolites integrated in the above results. Averaged ROC curves were generated by averaging the six polar metabolites ROC curves. ^*^ *p* < .05; ^**^ *p* < .01; ^***^ *p* < .001.

OPLS‐DA (orthogonal partial least squares discriminant analysis) is a statistical technique used for analysing multivariate data in order to identify the differences among different groups. It is commonly used in metabolomics and lipidomics research to identify biomarkers that are associated with different physiological or pathological conditions. We have used OPLS‐DA for the pairwise difference lipids analysis among study groups (Figure 2A). The results of the difference analysis are presented as a volcano plot. In the volcano plot, each metabolite or lipid is plotted as a point, its position on the *X*‐axis presents the magnitude of the change in the expression of the substance between the two groups, and its position on the *Y*‐axis presents the statistical significance of the change. This profile can be used to identify candidate metabolites or lipids that may be involved in specific biological processes or diseases. Volcano plots were generated by comparing the three groups populations in pairwise (Figure 2B). Combining results of Venn diagram (Figure 2C, VIP > 1, *p* < .05 and FC > 2 or <.5), four common differential lipids were selected for further study. These bar charts found that these four lipids were all increased in CRC patients (Figure 2E). ROC analysis of the four common differentially expressed lipids was performed to determine the diagnostic power between groups. Averaged ROC curves confirmed that four lipids had great diagnostic potential in CRC (Figure 2D). With these volcano plots, TAGs of adult CRC group were significantly increased as compared to HC (Figure 2F). This phenomenon was also observed in the volcano plot of senior CRC compared with HC (Figure 2G). A significant number of lipids were up‐regulated in adult CRC patients in comparison with that of senior CRC. Most of these lipids were belong to TAGs (Figure 2H).

**FIGURE 2:**
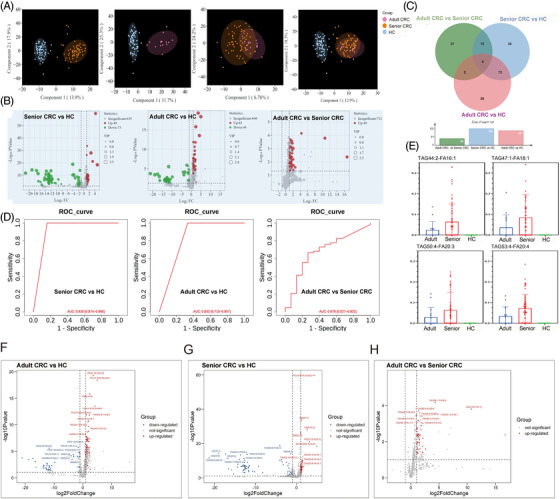
The lipidomics results of colorectal cancer (CRC) (A) orthogonal partial least squares discriminant analysis (OPLS‐DA) score plots were used for the pairwise difference polar metabolites analysis among adult CRC, senior CRC and healthy control (HC) groups. A distinct separation was revealed between adult CRC and HC groups. The distinct separation of senior CRC and HC groups also was observed. Adult and senior CRC groups did not separate by the OPLS‐DA model. (B) Volcano plots showed differences between pairwise comparisons of the three groups (adult CRC, senior CRC and HC). (C) Venn diagram to compare the similarities and differences of lipids between pairs of three groups. Combining the results with Venn diagram (variable influence on projection [VIP] > 1, *p* < .05 and FC > 2 or <.5), four common differential lipids were selected. (D) Averaged ROC curves (red) were generated by averaging these four lipids ROC curves. ROC analysis was applied using the four lipids integrated in the above results. These four lipids had the diagnostic potential between each of their pairwise groups. (E) These bar charts showed the expression levels of these four lipids in HC, adult CRC and senior CRC groups, respectively. The volcano plot in detail. (F–H) Volcano plots showed detailed differences among three groups (adult CRC vs. HC, adult CRC vs. senior CRC and senior CRC vs. HC, *p* < .05). The volcano plots annotated the top 10 most differentially expressed up‐ /down‐regulated lipids.

The correlation between polar metabolites and lipids was analysed by Spearman correlation test (Figure 3A). Network plot was used to illustrate these correlations (Figure 3B). By partitioning phenotypes, the lipid was correlated with laboratory examination (Figure 3C), pathognomonic symptoms (Figure 3D), signs (Figure 3D), symptoms (Figure 3E), underlying diseases (Figure 3E) and imaging results (Figure 3F). These results showed that lipids associated with clinical phenotypes were mostly TAGs, PCs and PEs. We further investigated the correlation of clinical phenoms and lipidomic results (Figure 3G).

**FIGURE 3:**
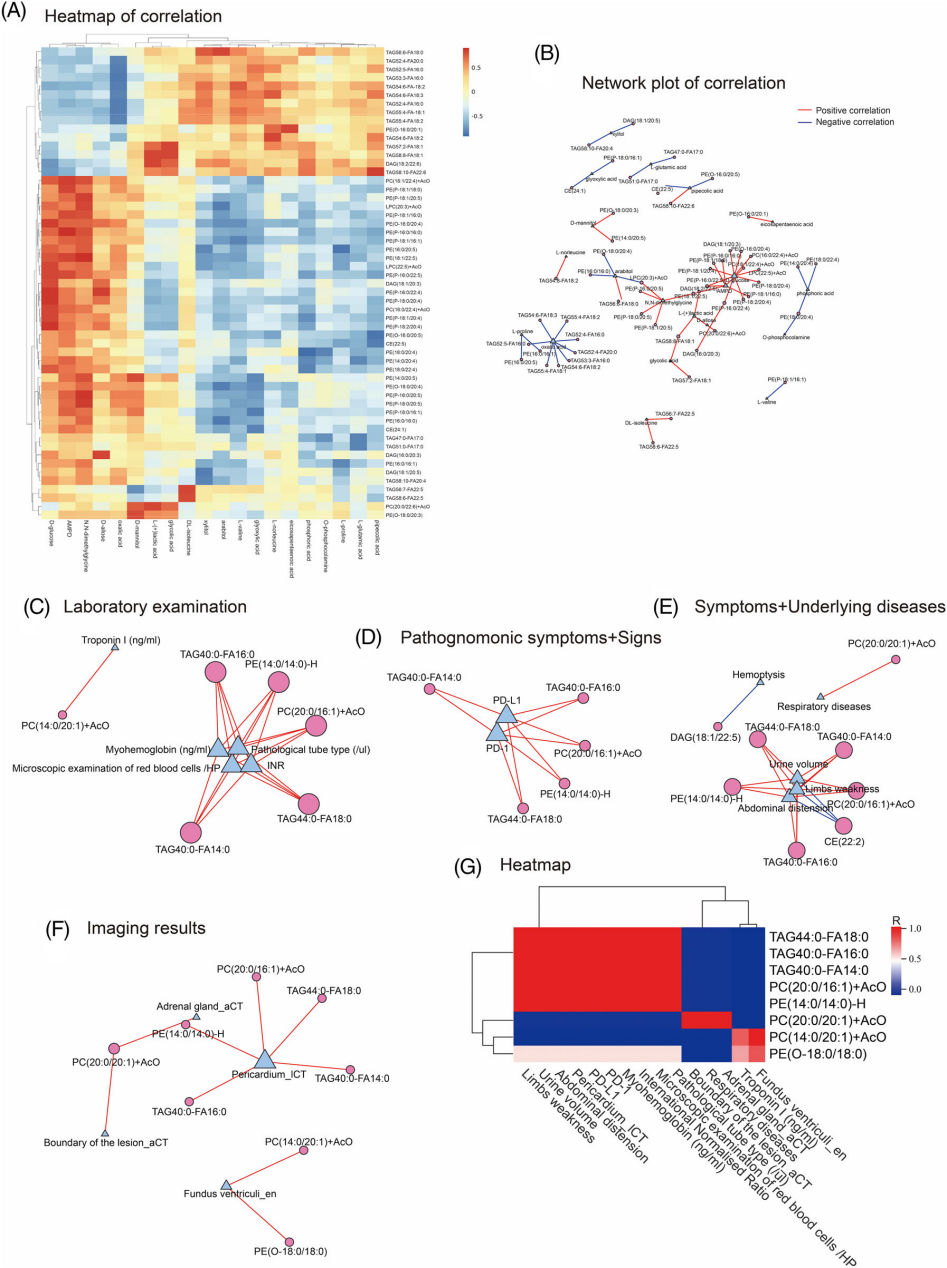
The interrelationship between the lipidomics and other omics, including the metabolomics and clinical phenotype. Heat map (A) and network plot (B) showing the associations between the changed polar metabolites and lipids. In the network plot, lines (red) mean positive correlations and lines (blue) mean negative correlations. Clinical phenotypes were separated into different types for analysis. The lipid‐phenotypic correlation was partitioned by laboratory examination (C), pathognomonic symptoms (D), signs (D), symptoms (E), underlying diseases (E) and imaging results (F). Lines (red) mean positive correlations and lines (blue) mean negative correlations. To better illustrate the correlation, all lipid‐related clinical phenotypes and their corresponding lipids were assembled and presented as heat map. All lipids associated with clinical phenotype were greater than 1 (G).

To evaluate the association between lipidomics profiles and patient gender, tumour markers and prognosis of human CRC, survival analysis using Log‐rank (Mantel–Cox) test explores the impact of lipids metabolism on CRC prognosis. CRC patients were followed up for up to 36 months. The effects of gender on CRC patients were determined by survival curve analysis and found that females had higher mortality and lower survival rate as compared to that of males (Figure 4A). Heat map was used to present the top 5 up‐regulated and 5 down‐regulated lipids in female group (VIP > 1, *p* < .05). In females, various CEs were higher than that of males, and various TAGs were lower. Grouped by tumour markers of plasma carcinoembryonic antigen (CEA), carcinoma antigen, including CA125, CA199 and CA724, the survival curves of CRC patients were analysed and differential lipids analysis performed as shown in Figure 4B–E. Results indicated that all these four common tumour markers can predict the survival of these patients. However, results of the lipidomics analysis after regrouping CRC patients by various tumour markers were different. For example, most of the up‐regulated lipids in CEA positive group were TAGs (mostly 46‐carbon), and most of the down‐regulated lipids were CEs, as compared to CEA negative group. Down‐regulations were seen in multiple SMs in the CA125 positive group, in comparison to negative group. The elevated lipids in CA199 positive group were all PEs, which did not appear in other tumour marker positive groups. Both up‐ and down‐regulated lipids in CA724 positive group were mostly TAGs, but the difference was that most of the up‐regulated TAGs were 54‐carbon TAGs, and most of the down‐regulated TAGs were 50‐carbon TAGs. Our results demonstrated that the combination of plasma lipids with tumour markers would not only be an effective biomarker for CRC, but also providing a new strategy for CRC diagnosis and monitoring.

**FIGURE 4:**
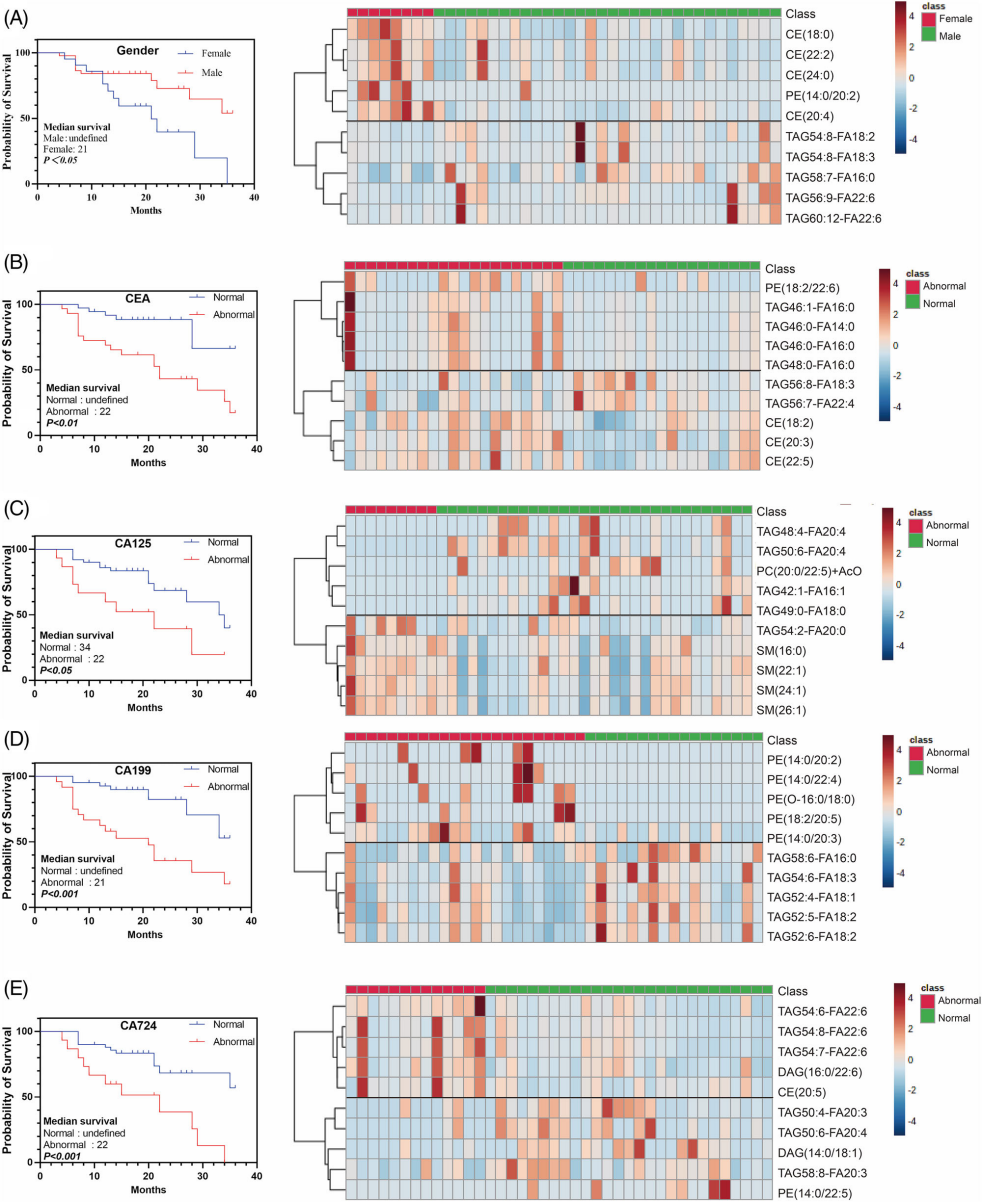
The impact of tumour markers on colorectal cancer (CRC) patient prognosis and their correlation with lipids. To evaluate the association between gender (A), tumour markers, including carcinoembryonic antigen (CEA) (B), CA125 (C), CA199 (D) and CA724 (E) and the overall survival in human CRC, survival analysis was operated by Log‐rank (Mantel–Cox) test, the impacts of lipids metabolism on prognosis were examined. CRC patients were followed up for up to 36 months, and their outcomes were obtained. The horizontal line in the middle of heat map, which represents the top 5 up‐ and down‐regulated lipids, is the dividing line between up‐ and down‐regulation lipids.

In conclusion, using an integrated trans‐omics approach, the study found that altered metabolites varied among genders, ages and clinical phenome. Relationships between lipids and polar metabolites, lipids and clinical phenotype, and lipids and multiple common tumour markers were also delineated. Furthermore, the study demonstrated the magnitude and complexity of phenome‐metabolic networks of CRC, which could facilitate the development of precision treatments that specifically target metabolites. The study underscored the great value of trans‐omics to uncover the heterogeneity of metabolism among CRC patients.

## CONFLICT OF INTEREST STATEMENT

The authors declare that they have no conflict of interests.

## Supporting information

Supporting Information

## Data Availability

The datasets used and/or analysed during the current study are available from the corresponding author on reasonable request.
