# Supplementary material for: Trans‐omic analyses identified novel prognostic biomarkers for colorectal cancer survival
Source: Clin Transl Med. 2023 Jun 16;13(6):e1304. doi: 10.1002/ctm2.1304 (PMC10276188; doi:10.1002/ctm2.1304)
Supplement: Supplementary file 1 — Supporting Information [file CTM2-13-e1304-s001.docx]

**SUPPLEMENTARY MATERIAL FOR**

**Trans-omic analyses identified novel prognostic biomarkers for colorectal cancer survival**

**Methods**

**Patient cohort**

All adult patients with pathologically confirmed diagnosis of CRC who admitted to the department of oncology from March 2019 to July 2021, and followed up until February 2022 were enrolled, with their plasma samples collected along with corresponding clinical data, which includes basic information, clinical manifestation, disease history, objective signs, laboratory results, radiological imaging, and histopathologic reports. The exclusion criteria were as follows: 1. Benign resections, i.e., polyps; 2. Prior history of cancers other than CRC; 3. With co-existing of any other malignant disorders.

Age- and gender-matched volunteers who went through physical examination to exclude CRC and other malignant diseases were enrolled as healthy controls (HC), during the same study period. The study was approved by the Institutional Review Board of Jinshan Hospital of Fudan University (# IEC-2020-S34). Written informed consents were obtained from all participants.

**Digital evaluation score system**

The Digital Evaluation Score System (DESS)^32^ is a clinical score index system, by which CRC patients were digital analyzed and evaluated as per descriptive severity information of each clinical phenome, and scored as 0, 1, 2, or 4: 4 standing for the most severe range, 2 indicating moderate condition, 1 representing mild or slight status, whereas 0 equating with normal or none (Table. S2).

**Metabolomics and lipidomics profiling**

The internal standard with mass spectrometry grade was tridecanoic acid (Sigma-Aldrich, St. Louis, MO, USA) in the polar metabolites detection part, while the Internal Standards Kit for Lipidyzer™ Platform (SCIEX, MA, USA) in the lipids detection part, respectively. The chemicals and reagents with mass spectrometry grade were subscribed from Fisher Chemical (Thermo Fisher, USA) or Sigma-Aldrich (St. Louis, MO, USA), including water, chloroform (CHCl_3_), methanol, acetonitrile, isopropanol, pyridine, acetone, ammonium hydroxide solution, ammonium acetate(NH4OAc), methoxyamine hydrochloride, and N-methyl-N-(trimethylsilyl)trifluoroacetamide(MSTFA)^28,33^.

For polar metabolites extraction, 100ul plasma of each sample was used. Quality control (QC) samples were prepared by combining equal aliquots of samples. 400 ul solution (ratio of volume, methanol: water = 4:1, containing 5ug/ml tridecanoic acid as the internal standard) was added to plasma samples and QC. The mixtures were vigorously mixed at 37°C for 30 s and then gently shaken at 1200 rpm for 30 min. Then the mixtures were centrifuged at 14,000 rpm for 15 min at 4 °C and the supernatant was collected. The supernatants were concentrated, frozen and freeze-dried. We used two-step derivatization protocol for subsequent extraction. The samples were resuspended in 50 μl of 20 mg/ml methoxyamine hydrochloride in pyridine. The tubes were vortexed, sonicated for one minute and then incubated with shaking at 30 °C for 90 min to form methoxyamine derivatives. Subsequently, 40 μl of MSTFA was added to the sample for silylation reaction and incubated in 37 °C for 30 min. The supernatants were collected after centrifugation at 14000 rpm for 5 min. The supernatant was collected and placed in a sample tube awaiting GC-MS analysis. Untargeted metabolomics analysis was carried out by gas chromatography-mass spectrometry (GC-MS) with an Agilent 7890B GC and 5977B inert mass selective detector (MSD) system (Agilent Technologies, Santa Clara, CA, USA). The raw data from GC-MS was performed using Agilent MassHunter Qualitative Analysis software (version 10.0, Agilent, CA, USA). The Agilent Fiehn database was referred to for identification of the metabolites^34^. By using the formula: standard sample peak area/standard sample concentration = sample peak area/sample concentration, the metabolites content in the sample was calculated^35^.

For lipids extraction, Add 350ul 4 °C isopropanol and 9ul internal standard cocktails to each sample of 20ul plasma or QC reagents. Mix 1 minute and incubate 10 min at room temperature, then store overnight at −20 °C. On the 2nd day, centrifuge 12,000 rpm 20 min, collect 200 μL supernatant and store at −80 °C. We performed plasma Lipidomics at AB SCIEX QTRAP 5500 LC-MS/MS system. The extracted Lipid samples were injected into Waters Acquity UPLC BEH HILIC column (100 mm×2.1 mm,1.7µm) with a Waters Acquity UPLC BEH HILIC VanGuard Pre column (2.1mm×5mm, 1.7um,). A phase was 95% acetonitrile (acetonitrile: water, V: V, 95:5) containing with 10 mmol/L ammonium acetate, and B phase was 50% acetonitrile (acetonitrile: water, V: V, 50:50) containing with 10 mmol/L ammonium acetate. The ammonium hydroxide was added to the B phase until its PH is equal to the A phase pH. The flow rate was 0.5mL/min. A gradient elution was performed as follows, B phase started with 0.1% and increased to 20% during 10 min, then linearly increased to 98% during 10min to 11 min, and 98% of B phase was held for 2 mins, and returning to the initial conditions 0.1% in 13.1 min. The 0.1% of B phase was held until 16 min. The positive electrospray ionization (ESI+) mode injection volume was 2 μL, and the negative electrospray ionization (ESI–) mode injection volume was 5 μl. The N2 was used as dissolvent. Parameter settings were as follows, curtain gas: 35psi, GS1: 50psi, GS2: 60psi, ion spray voltage is: 5500V, declustering potential: 80V, entrance energy: 10V, collision energy:50V. Data were acquired using the Analyst software (version 1.7, SCIEX, MA, USA). MultiQuant software (AB SCIEX) was used to process data, after lipids were identified by mass spectrometry.

**Comprehensive analyses of trans-omics**

Metabo Analyst software 4.0 (www.metaboanalyst.ca) was utilized for conducting multivariate statistical analysis, cluster analysis, dimensionality reduction, and making heat map. Correlations between different omics data were calculated using Spearman’s rank correlation.

**Statistical analysis**

Data were presented as mean ± SE or median with interquartile range (IQR). The means of each group were used for calculation and comparison. Statistical significance of differences between two groups or among multiple groups was determined by Student’s t-test, or one-way ANOVA test, respectively. The *p-*value were adjusted for the type I error rate in multiple comparisons. Statistical significance was affirmed when *p-*value<0.05. The potential biomarkers were selected based on VIP values greater than 1. Survival curves were assessed through Log-rank (Mantel-Cox) test. The average ROC curve was plotted through <http://www.bioinformatics.com.cn/>. In addition, we also validated the metabolites or lipids using 5-fold cross-validation, we evaluated the model in the test and training cohort in Fig. S1.

**Figure S1. ROC curve of 5-fold cross-validation.**

The average ROC curves in the metabolomics results were performed by 5-fold cross-validation (A, B, C). The average ROC curves in the lipidomics results section performed by by 5-fold cross validation (D, E, F).

| **Table S1. Clinical characteristics of CRC patients** | | | |
| --- | --- | --- | --- |
|  | **CRC patients in corhort 1(n=78)** | **CRC patients in corhort 2(n=31)** | ***p-*value** |
| **Detection condition** |  |  |  |
| Sample type | Plasma | Plasma |  |
| Method | LC-MS | GC-MS |  |
| Result | Lipids | Polar metabolites |  |
| **Clinical Characteristics** |  |  |  |
| Age (Median, years) | 68 | 66 | 0.708 |
| Gender (number, percent) | Male(49, 62.8%), Female(29, 37.2%) | Male (22, 71.0%), Female (9, 29.0%) | 0.135 |
| Groupe by age (A: adult, S: senior) | Y:15, S:63 | Y:8, S:23 | 0.448 |
| **Stage** |  |  | 0.297 |
| I | 1 | 1 |  |
| II | 6 | 0 |  |
| III | 12 | 2 |  |
| IV | 21 | 10 |  |
| **Treatment (by age group)** |  |  | 0.616 |
| Surgery | 38 | 12 |  |
| Chemotherapy | 38 | 12 |  |
| Radiation | 12 | 9 |  |
| Immunotherapy | 7 | 2 |  |
| Targeted therapy | 26 | 12 |  |
| Neoadjuvant chemotherapy | 9 | 3 |  |
| **Complication (by age group)** |  |  |  |
| Ileus | 1 | 1 | 0.802 |
| Enterobrosis | 1 | 0 |  |
| **Outcome** |  |  | 0.769 |
| alive | 63 | 24 |  |
| dead | 15 | 7 |  |

**Table S2. Clinical phenomes scored in lung cancer patients**

| Clinical phenomes | DESS Scores | | | |
| --- | --- | --- | --- | --- |
|  | 0 | 1 | 2 | 4 |
| Basic information | | | | |
| Age | <40 | 40-49 | 50-74 | ≥75 |
| Length of stay | <1 | 1-3 | 4-7 | >7 |
| Symptoms | | | | |
| Shiver | no | slight | medium | severe |
| Dizziness | no | slight | medium | severe |
| Headache | no | slight | medium | severe |
| Hyposarca | no | slight | medium | severe |
| Tired | no | slight | medium | severe |
| Fever | no | slight | medium | severe |
| Disturbance of consciousness | no | slight | medium | severe |
| Blurred vision | no | slight | medium | severe |
| Eye drying | no | slight | medium | severe |
| Tinnitus | no | slight | medium | severe |
| Amnesia | no | slight | medium | severe |
| Testiness | no | slight | medium | severe |
| Limitation of mouth opening | no | slight | medium | severe |
| Thirst | no | slight | medium | severe |
| Ozostomia | no | slight | medium | severe |
| Bitter taste | no | slight | medium | severe |
| Distortion of commissur | no | slight | medium | severe |
| Pharyngalgia | no | slight | medium | severe |
| Dysphagia | no | slight | medium | severe |
| Palpitation | no | slight | medium | severe |
| Stethalgia | no | slight | medium | severe |
| Chest distress | no | slight | medium | severe |
| Cough | no | slight | medium | severe |
| Expectoration | no | slight | medium | severe |
| Hemoptysis | no | slight | medium | severe |
| Shortness of breath | no | slight | medium | severe |
| Asthma | no | slight | medium | severe |
| Breathing difficulties | no | slight | medium | severe |
| Nausea | no | slight | medium | severe |
| Emesis | no | slight | medium | severe |
| Hemoptysis | no | slight | medium | severe |
| Acid reflux | no | slight | medium | severe |
| Bad appetite | no | slight | medium | severe |
| Abdominal distension | no | slight | medium | severe |
| Diarrhea | no | slight | medium | severe |
| Constipation | no | slight | medium | severe |
| Hematochezia | no | slight | medium | severe |
| Lumbodorsalgia | no | slight | medium | severe |
| Joint pain | no | slight | medium | severe |
| Frequency of urinatior | no | slight | medium | severe |
| Nocturia increased | no | slight | medium | severe |
| Odynuria | no | slight | medium | severe |
| Vesical tenesmus | no | slight | medium | severe |
| Hematuresis | no | slight | medium | severe |
| Uroclepsia | no | slight | medium | severe |
| Dysuresia | no | slight | medium | severe |
| Anemia | no | slight | medium | severe |
| Tumor-related pain | no | slight | medium | severe |
| Morning stiffness | no | slight | medium | severe |
| Joint swelling | no | slight | medium | severe |
| Limbs weakness | no | slight | medium | severe |
| Intermittent claudication | no | slight | medium | severe |
| Rest pain | no | slight | medium | severe |
| Limbs numbness | no | slight | medium | severe |
| Limbs pain | no | slight | medium | severe |
| Limbs cold | no | slight | medium | severe |
| Muscle spasm | no | slight | medium | severe |
| Muscle tremors | no | slight | medium | severe |
| Sleeping | no | slight | medium | severe |
| Night sweat | no | slight | medium | severe |
| Marasmus | no | slight | medium | severe |
| Obesity | no | slight | medium | severe |
| Tetter | no | slight | medium | severe |
| Ecchymosis | no | slight | medium | severe |
| Underlying diseases | | | | |
| Hypertension | no | ≤5 years | 5-10 years | ≥10 years |
| Diabetes | no | ≤5 years | 5-10 years | ≥10 years |
| High blood pressure | no | Level 1 | Level 2 | Level 3 |
| Hypertension medication | no | 1 treatment | 2 treatments | 3 or more treatments |
| Hypertension stratification | low-risk | slight-risk | medium-risk | severe-risk |
| Oral hypoglycemic drug | no | 1 treatment | 2 treatments | 3 or more treatments |
| hyperlipidaemia | no | ≤5 years | 5-10 years | ≥10 years |
| hyperuricemia | no | ≤5 years | 5-10 years | ≥10 years |
| Cerebral infarction | no | ≤5 years | 5-10 years | ≥10 years |
| Chronic lung disease | no | ≤5 years | 5-10 years | ≥10 years |
| Hypothyroidism | no | ≤5 years | 5-10 years | ≥10 years |
| Hyperthyroidism | no | ≤5 years | 5-10 years | ≥10 years |
| Nephropathy | no | ≤5 years | 5-10 years | ≥10 years |
| Autoimmune disease | no | ≤5 years | 5-10 years | ≥10 years |
| Coronary heart disease | no | ≤5 years | 5-10 years | ≥10 years |
| Coronary heart disease treatment | no | aspirin | multiple drugs | heart stent |
| Peptic ulcer | no | ≤5 years | 5-10 years | ≥10 years |
| Chronic liver disease | no | ≤5 years | 5-10 years | ≥10 years |
| Liver cirrhosis | no | ≤5 years | 5-10 years | ≥10 years |
| Cerebrovascular disease | no | ≤5 years | 5-10 years | ≥10 years |
| Other cancers | | | | |
| Nasopharynx cancer | no |  |  | yes |
| Laryngeal cancer | no |  |  | yes |
| Gastric cancer | no |  |  | yes |
| Liver cancer | no |  |  | yes |
| Pancreatic cancer | no |  |  | yes |
| Esophagus cancer | no |  |  | yes |
| Thyroid cancer | no |  |  | yes |
| Breast cancer | no |  |  | yes |
| Colorectal cancer | no |  |  | yes |
| Renal cancer | no |  |  | yes |
| Cerebral cancer | no |  |  | yes |
| Prostatic cancer | no |  |  | yes |
| Testicular cancer | no |  |  | yes |
| Ovarian cancer | no |  |  | yes |
| Cervical cancer | no |  |  | yes |
| Uterus Cancer | no |  |  | yes |
| Metastasis | | | | |
| Brain metastases | no |  |  | yes |
| Osseous metastasis | no |  |  | yes |
| Hepatic metastases | no |  |  | yes |
| Adrenal metastasis | no |  |  | yes |
| Renal metastasis | no |  |  | yes |
| Chest metastasis | no |  |  | yes |
| Pleural metastasis | no |  |  | yes |
| Skull metastasis | no |  |  | yes |
| Lymphatic metastasis | no |  |  | yes |
| Personal history | | | | |
| Smoking | never | cessation | occasionally | everday |
| Pack-years of smoking | 0 | <20 | 20-40 | ≥40 |
| Drink | never | cessation | occasionally | everday |
| Pack-years of drinking | 0 | <20 | 20-40 | ≥40 |
| Drug allergy history | no |  |  | yes |
| Operation History | no |  |  | yes |
| Family history | no |  |  | yes |
| Physical examination | | | | |
| Breathe(Times/minutes) | 12-20 | 9-11 | 21-24 | ≤8 or ≥25 |
| Temperature(℃) | 36.1-37.3 | 35.1-36.0 or 37.4 -39.0 | ≥39.1 | ≤35.0 or ＞41 |
| Heart rate(Times/minutes) | 60-90 | 41-59 or 91-110 | 111-130 | ≤40 or ≥131 |
| Systolic pressure(mmHg) | 110-130 | 131-139 or  101-109 | 140-170 or  90-100 | >170 or <90 |
| Diastolic pressure(mmHg) | 60-80 | 80-89 | ≥90 |  |
| General condition | | | | |
| Mental condition | no | slight | medium | severe |
| Nutritional state | no | slight | medium | severe |
| Physical development | no | slight | medium | severe |
| Face | no | slight | medium | severe |
| Hair | no | slight | medium | severe |
| Skin | no | slight | medium | severe |
| Superficial lymph node | no | slight | medium | severe |
| Eyesight | no | slight | medium | severe |
| Skull deformity | no | slight | medium | severe |
| Hearing | no | slight | medium | severe |
| Hoarseness | no | slight | medium | severe |
| Cyanosis | no | slight | medium | severe |
| Dental ulcer | no | slight | medium | severe |
| Aphasia | no | slight | medium | severe |
| Distention of jugular vein | no | slight | medium | severe |
| Thoracocyllosis | normal |  |  | abnormal |
| Intercostal change | normal |  |  | abnormal |
| Three depressions sign | normal |  |  | abnormal |
| Sense pleural friction | normal |  |  | abnormal |
| Moist rales | no | slight | medium | severe |
| Wheezes | no | slight | medium | severe |
| Velcro | no | slight | medium | severe |
| Voice transmission | no | slight | medium | severe |
| Cardiac souffle | no | slight | medium | severe |
| Pericardial friction rub | no |  |  | yes |
| Peripheral vascular examination | normal |  |  | abnormal |
| Abdominal mass | no |  |  | yes |
| Abdominal respiration | no |  |  | yes |
| Abdominal varicose veins | no |  |  | yes |
| Abdominal tenderness | no |  |  | yes |
| Murphy sign | no |  |  | yes |
| Hepatomegaly | no |  |  | yes |
| Splenomegaly | no |  |  | yes |
| Bowel sound | no |  |  | yes |
| Liver area knocking pain | no |  |  | yes |
| Renal area knocking pain | no |  |  | yes |
| Shifting dullness | no |  |  | yes |
| Ataxia | no |  |  | yes |
| Clubbing finger | no |  |  | yes |
| Ankylosis | no | slight | medium | severe |
| Redness and swelling of joints | no | slight | medium | severe |
| Joint tenderness | no | slight | medium | severe |
| Limb deformities | no |  |  | yes |
| Amyotrophy | no |  |  | yes |
| Lower limb edema | no | slight | medium | severe |
| Anaesthesia | no | slight | medium | severe |
| Muscular tension | normal |  |  | abnormal |
| Corneal reflex | normal |  |  | abnormal |
| Abdominal reflexes | normal |  |  | abnormal |
| Tendon reflex | normal |  |  | abnormal |
| Patellar tendon reflex | normal |  |  | abnormal |
| Achilles tendon reflex | normal |  |  | abnormal |
| Hoffmann sign | normal |  |  | abnormal |
| Babinski sign | normal |  |  | abnormal |
| Oppenheim sign | normal |  |  | abnormal |
| Kernig sign | normal |  |  | abnormal |
| Brudzinski sign | normal |  |  | abnormal |
| Pathological examination | | | | |
| Pathology grade(WHO) | no | I | II | III |
| Maximum tumor diameter | no | >2 | 2-4 | >4 |
| Differentiation | precancerous lesions | Well differentiated | moderately differentiated | poorly differentiated |
| Primary site | no | 1 | 2 | >2 |
| Number of primary lesions | no | 1 | 2 | >2 |
| Number of lymph nodes involved | no | 1-2 | 3-5 | >5 |
| EBER | negative |  |  | positive |
| EMA | negative |  |  | positive |
| ER | negative |  |  | positive |
| PR | negative |  |  | positive |
| HER-2 | negative |  |  | positive |
| GFAP | negative |  |  | positive |
| Olig-2 | negative |  |  | positive |
| BRAF | negative |  |  | positive |
| K-ras | negative |  |  | positive |
| Vimentin(VIM) | negative |  |  | positive |
| S-100 | negative |  |  | positive |
| D2-40 | negative |  |  | positive |
| CD34 | negative |  |  | positive |
| CD56 | negative |  |  | positive |
| CDX2 | negative |  |  | positive |
| Cam5.2 | negative |  |  | positive |
| CgA | negative |  |  | positive |
| P53 | negative |  |  | positive |
| MC | negative |  |  | positive |
| NapsinA | negative |  |  | positive |
| NF | negative |  |  | positive |
| calretinin | negative |  |  | positive |
| β-catenmin | negative |  |  | positive |
| cyclin D1 | negative |  |  | positive |
| IDH1 | negative |  |  | positive |
| SOX10 | negative |  |  | positive |
| Syn | negative |  |  | positive |
| PTEN | negative |  |  | positive |
| PD-1 | negative |  |  | positive |
| PD-L1 | negative |  |  | positive |
| EGFR | negative |  |  | positive |
| T790 | negative |  |  | positive |
| ALK | negative |  |  | positive |
| ROS | negative |  |  | positive |
| CK | negative |  |  | positive |
| CK5/6 | negative |  |  | positive |
| CK7 | negative |  |  | positive |
| CK8/18 | negative |  |  | positive |
| CK14 | negative |  |  | positive |
| CK20 | negative |  |  | positive |
| P63 | negative |  |  | positive |
| P40 | negative |  |  | positive |
| LCA | negative |  |  | positive |
| Ki-67 | negative |  |  | positive |
| T(tumor) | Tx and T1 | T2 | T3 | T4 |
| N(lymph node) | no | N1 | N2 | N3 |
| Metastasis | Mx and M0 |  |  | M1 |
| Imageological examination | | | | |
| Heart | normal |  |  | abnormal |
| Pericardium | normal |  |  | abnormal |
| Lung | normal |  |  | abnormal |
| Mass number | 0 | 1 | 2 | >2 |
| Tumor diameter | no | <1cm | 1-2cm | >2cm |
| Hilar | normal |  |  | abnormal |
| Mediastinum | normal |  |  | abnormal |
| Lymphadenectasis | 0 | 1-2 | 3-5 | >5 |
| Hydrothorax | 0 | <300ml | 300-500ml | >500ml |
| Emphysema | no |  |  | yes |
| Lung texture change | normal |  |  | abnormal |
| Active lesions | normal |  |  | abnormal |
| Old lung lesions | normal |  |  | abnormal |
| Electrocardiogram sign | | | | |
| Sinus rhythm | normal |  |  | abnormal |
| Conduction block | normal |  |  | abnormal |
| Atrial fibrillation | normal |  |  | abnormal |
| Ventricular premature contraction | normal |  |  | abnormal |
| Atrial premature contractions | normal |  |  | abnormal |
| P-wave | normal |  |  | abnormal |
| QRS | normal |  |  | abnormal |
| ST segment | normal |  |  | abnormal |
| T-wave | normal |  |  | abnormal |
| Q-T interval | normal |  |  | abnormal |
| Abdomen ultrasound | | | | |
| Liver | normal |  |  | abnormal |
| Pancreas | normal |  |  | abnormal |
| Spleen | normal |  |  | abnormal |
| Cholecyst | normal |  |  | abnormal |
| Adrenal gland | normal |  |  | abnormal |
| kidney | normal |  |  | abnormal |
| Ureter | normal |  |  | abnormal |
| Bladder | normal |  |  | abnormal |
| Abdominal lymph node | 0 | 1-2 | 3-5 | >5 |
| Lung CT | | | | |
| Trachea and bronchus | normal |  |  | abnormal |
| Pleura | normal |  |  | abnormal |
| Chest wall soft tissue | normal |  |  | abnormal |
| Coronary artery | normal |  |  | abnormal |
| Lung consolidation | normal |  |  | abnormal |
| Pulmonary embolism | normal |  |  | abnormal |
| Post enhancement | normal |  |  | abnormal |
| Lesions progress | normal |  |  | abnormal |
| Lung function | | | | |
| Restricted ventilation dysfunction | no | slight | medium | severe |
| Obstructive ventilation function disturbance | no | slight | medium | severe |
| Oxygen saturation(%) | no | slight | medium | severe |
| Vital capacity | no | slight | medium | severe |
| FEVI | no | slight | medium | severe |
| MEFV | no | slight | medium | severe |
| Cardiac ultrasound | | | | |
| EF (%) | 50-75 | 40-50 | 30-40 | <30 |
| E/E' | <8 | E/A>1 | E/A<1 | >15 |
| PASP(mmHg) | ≤30 | 30-40 | 40-50 | >50 |
| LVH | normal |  |  | abnormal |
| LVDF | normal |  |  | abnormal |
| Aortic regurgitation | no | slight | medium | severe |
| Pulmonary regurgitaion | no | slight | medium | severe |
| Calcification | no | slight | medium | severe |
| Head CT | | | | |
| Face | normal |  |  | abnormal |
| Maxillofacial | normal |  |  | abnormal |
| Neck | normal |  |  | abnormal |
| Pituitary gland | normal |  |  | abnormal |
| Frontal lobe | normal |  |  | abnormal |
| Parietal lobe | normal |  |  | abnormal |
| Temporal lobe | normal |  |  | abnormal |
| Occipital lobe | normal |  |  | abnormal |
| Pons | normal |  |  | abnormal |
| Cerebellar hemisphere | normal |  |  | abnormal |
| Sulci and gyri | normal |  |  | abnormal |
| Ventricle and cisterna lesions | normal |  |  | abnormal |
| Signal uniformity | normal |  |  | abnormal |
| Cerebral hernia | normal |  |  | abnormal |
| Focal ischemia | no | slight | medium | severe |
| MRI examination | | | | |
| Cervical | normal |  |  | abnormal |
| Thoracic | normal |  |  | abnormal |
| Lumber | normal |  |  | abnormal |
| Sacral | normal |  |  | abnormal |
| Neck 3-4 | normal |  |  | abnormal |
| Neck 4-5 | normal |  |  | abnormal |
| Neck 5-6 | normal |  |  | abnormal |
| Neck 6-7 | normal |  |  | abnormal |
| Lumbar 1-2 | normal |  |  | abnormal |
| Lumbar 2-3 | normal |  |  | abnormal |
| Lumbar 3-4 | normal |  |  | abnormal |
| Lumbar 4-5 | normal |  |  | abnormal |
| Lumbar 5-sacral 1 | normal |  |  | abnormal |
| Sacral 1-5 | normal |  |  | abnormal |
| Caudal 1-3 | normal |  |  | abnormal |
| Abdominal CT | | | | |
| Stomach | normal |  |  | abnormal |
| Perigastric lymph node | normal |  |  | abnormal |
| Intrahepatic bile duct | normal |  |  | abnormal |
| Pancreatic duct | normal |  |  | abnormal |
| Epityphlon | normal |  |  | abnormal |
| Intestinal wall | normal |  |  | abnormal |
| Enteric cavity | normal |  |  | abnormal |
| Seroperitoneum | normal |  |  | abnormal |
| Urine/stool routines | | | | |
| Urine color | normal |  |  | abnormal |
| Urine transparency | - | ± | + | ≥++ |
| Urine PH | 4.5-8.0 |  |  | <4.5 or >8.0 |
| Urine specific gravity | 1.000-1.030 |  |  | <1.000 or >1.030 |
| Urine glucose | - | ± | + | ≥++ |
| Urine protein | - | ± or + | ++ | >++ |
| Urobilirubin | - | ± | + | ≥++ |
| Urobilinogen | - | ± | + | ≥++ |
| Urine acetone bodies | ± | + | ++ | ≥+++ |
| Urinary nitrite | - | ± | + | ≥++ |
| Urine occult blood | - | ± | + | ≥++ |
| Urine leukocyte (/ul) | - | ± | + | ≥++ |
| Urinary vitamin C | - | ± | + | ≥++ |
| Microscopic examination of red blood cells /HP | 0-3 | >3 | >5 | >8 |
| Microscopic examination of white blood cell /HP | 0-5 | >5 | >10 | >25 |
| Microscopic examination of crystallization number | - | ± | + | ≥++ |
| Urine erythrocyte (/ul) | 0-10 | 10.1-50.0 | 50.1-100.0 | >100.0 |
| Urinary non-squamous epithelial cells (/ UL) | 0-2 | >2 | >6 | >10 |
| - Urinary squamous epithelial cells (/ul) | 0-5 | >5 | >15 | >25 |
| Transparent tube type (/ul) | 0-2 |  |  | >2 |
| - Pathological tube type (/ul) | 0-0.5 |  |  | >0.5 |
| Yeast (/ ul) | 0-3 |  |  | >3 |
| Stool color | normal |  |  | abnormal |
| Stool property | normal |  |  | abnormal |
| Mucus | normal |  |  | abnormal |
| Fecal occult blood | normal |  |  | abnormal |
| Chemical measurements | | | | |
| AFP (ng/ml) | 0-9 | <200 | > 200 | > 400 |
| AFP-L3/AFP(%) | 0-10 | <3 xULN | >3-5 xULN | >5 xULN |
| CEA (ng/ml) | 0-5 | <3 xULN | >3-5 xULN | >5 xULN |
| CA242 (U/ml) | 0-25 | <3 xULN | >3-5 xULN | >5 xULN |
| CA50 (U/ml) | 0-25 | <3 xULN | >3-5 xULN | >5 xULN |
| CA199 (U/ml) | 0-25 | <3 xULN | >3-5 xULN | >5 xULN |
| CA125 (U/ml) | 0-35 | <3 xULN | >3-5 xULN | >5 xULN |
| CA153 (U/ml) | 0-14 | <3 xULN | >3-5 xULN | >5 xULN |
| CA724 (U/L) | 0-6.9 | <3 xULN | >3-5 xULN | >5 xULN |
| SCCA (ng/ml) | 0-1.5 | <3 xULN | >3-5 xULN | >5 xULN |
| NSE (ng/ml) | 0-16.3 | <3 xULN | >3-5 xULN | >5 xULN |
| CYFRA21-1(ng/ml) | 0-3.3 | <3 xULN | >3-5 xULN | >5 xULN |
| PSA (ng/ml) | 0-4 | <3 xULN | >3-5 xULN | >5 xULN |
| FPSA/TPSA (%) | ＞0.26 |  |  | beyond normal limits |
| PAP (ng/ml) | 0-2 | <3 xULN | >3-5 xULN | >5 xULN |
| Ferritin (ng/ml) | 30-400 | <3 xULN | >3-5 xULN | >5 xULN |
| Thyroglobulin (ng/ml) | 3.5-77 | <2 xULN | >2 xULN | >5 xULN |
| Thyroglobulin antibody (IU/ml) | 0-115 | <2 xULN | >2 xULN | >5 xULN |
| Anti-thyroid peroxidase antibody (IU/ml) | 0-34 | <2 xULN | >2 xULN | >5 xULN |
| Thyrotropin receptor antibody (IU/L) | 0-1.75 | <2 xULN | >2 xULN | >5 xULN |
| T4 (nmol/L) | 66-181 | <2 xULN | >2 xULN | >5 xULN |
| fT4 (pmol/L) | 12.0-22 | <2 xULN | >2 xULN | >5 xULN |
| T3 (nmol/L) | 1.3-3.1 | <2 xULN | >2 xULN | >5 xULN |
| fT3 (pmol/L) | 3.1-6.8 | <2 xULN | >2 xULN | >5 xULN |
| TSH (mIU/L) | 0.27-4.2 | <2 xULN | >2 xULN | >5 xULN |
| IgG (g/L) | 7.0-16 | <2 xULN | >2 xULN | >5 xULN |
| IgG4 (g/L) | 0.03-2.01 | <2 xULN | >2 xULN | >5 xULN |
| IgA (g/L) | 0.7-4 | <2 xULN | >2 xULN | >5 xULN |
| IgM (g/L) | 0.4-2.3 | <2 xULN | >2 xULN | >5 xULN |
| IgE (IU/mL) | 0-100 | <2 xULN | >2 xULN | >5 xULN |
| Complement C3 (g/L) | 0.9-1.8 | <2 xULN | >2 xULN | >5 xULN |
| Complement C4 (g/L) | 0.1-0.4 | <2 xULN | >2 xULN | >5 xULN |
| Transferrin (g/L) | 2-3.6 | <2 xULN | >2 xULN | >5 xULN |
| Ceruloplasmin (g/L) | 0.2-0.6 | <2 xULN | >2 xULN | >5 xULN |
| PH | 7.35-7.45 | 7.29-7.36 or 7.46-7.49 | 7.2-7.3 or 7.5-7.6 | <7.2 or >7.6 |
| PaO2 (mmHg) | ≥90 | 60-90 | 40-60 | <40 |
| PaCO2 (mmHg) | 35-45 | 46-49 or 29-34 | 50-70 or 20-30 | <20 or >70 |
| SaO2 (%) | ≥95 | 86-94 | 75-85 | <75 |
| PT (s) | 11-17.8 | ULN-20.8 (ULN+3) | 20.9-30 | <9s or >30s |
| APTT(s) | 25.4-38.4 | ULN-44.9 or LLN-19.9 | - 1. or 16-20 | >60s or <16s |
| Fibrinogen (g/L) | 2.0-4 | <2 or >4 | <1.5 or 5.5 | <1 or >6 |
| TT(s) | 11-17.8 | 17.9-29.9 | 30-60 | >60s |
| AT-A (%) | 75.6-113.4 | <2 xULN | >2 xULN | >5 xULN |
| FDP (mg/L) | 0-5 | 4.9-14.9 | 15-40 | >40 |
| D-Dimer (mg/L) | 0-0.232 | >0.232 | >0.5 | >1 |
| INR | ≤ XULN | <3 | 3.0-3.5 | >3.5 |
| HBVe Ag | negative |  |  | positive |
| HBVe Ab | negative |  |  | positive |
| HBVs Ag | negative |  |  | positive |
| HBVs Ab | negative |  |  | positive |
| HBVc Ab | negative |  |  | positive |
| HBVc Ab IgM | negative |  |  | positive |
| HCV Ab IgG | negative |  |  | positive |
| HEV Ab IgG | negative |  |  | positive |
| HEV Ab IgM | negative |  |  | positive |
| HBV DNA copy number | negative |  |  | positive |
| Treponema pallidum specific antibody | negative |  |  | positive |
| TRUST | negative |  |  | positive |
| HPV | negative |  |  | positive |
| HIV | negative |  |  | positive |
| EB | negative |  |  | positive |
| HP | negative |  |  | positive |
| Total cholesterol (mmol/L) | 3-5.7 | 5.7-7 | 7-8 | >8 |
| Triglyceride (mmol/L) | 0-1.7 | 1.7-3.0 | 3.0-4.0 | >4 |
| HDL (mmol/L) | 1.03-2.07 | 0.91-2.07 |  | ≤0.91 |
| LDL (mmol/L) | ≤3.12 | 3.12-3.16 | 3.16-3.64 | >3.64 |
| Glycosylated hemoglobin (%) | 4-6 | 6-8 | 8-9 | >9 |
| Fasting blood-glucose (mmol/L) | 4.1-5.9 | 5.9-7 | 2.8-4.1 or 7-24.8 | <2.8 or >24.8 |
| Glomerular filtration rate (ml/min*1.73m2) | ≥90 | 60-89 | 30-59 | <30 |
| ALB | 1.2-2.4 |  | beyond normal limits |  |
| Albumin (g/L) | 35-55 or normal | <35 or >55 | <28 or >60 | <20 or >80 |
| Total protein (g/L) | 65-85 | <65 or >85 | <50 or >90 | <30 or >100 |
| ALT (U/L) | 9-50 | <9 or >50 | >200 | >500 |
| AST (U/L) | 15-40 | <15 or >40 | >200 | >500 |
| ALP(U/L) | 45-125 | < 2 xULN | >2 xULN | >5 xULN |
| Lactic dehydrogenase (U/L) | 120-250 | >250 or <120 | >500 | >1000 |
| Cystatin C(mg/L) | 0.6-1.3 | <0.6 or 1.3-2.63 | 2.64-4.90 | >4.9 |
| Gamma-GT (U/L) | 10-60 | < 2 xULN | >2 xULN | >5 xULN |
| Total bilirubin (umol/L) | <34.2 | 34.2-171 | 171-342 | >342 |
| Direct bilirubin (umol/L) | <3.4 | 3.4-17.1 | 17.1-34.2 | >34.2 |
| Urea (mmol/L) | 3.2-7.1 | 7.2-9 or <3.2 | 9-20 | >20 |
| Creatinine (umol/L) | 58-110 | <450 | <707 | >1500 |
| Uric Acid (umol/L) | ≤420 | >420 | >600 | >750 |
| β2-MG (mg/L) | 0-2.7 | < 2 xULN | >2 xULN | >5 xULN |
| Na(mmol/L) | 137-145 | 146-155,or125-136 | 155-160 or 120-124 | >160 or <120 |
| K(mmol/L) | 3.5-5.1 | 3.2-3.4 | 2.5-3.1/5.2-6.1 | <2.5 or >6.2 |
| Cl(mmol/L) | 98-107 | <98 or >107 | <95 or >115 | <90 or >120 |
| Ca(mmol/L) | 2.1-2.55 | 1.74-2.09 or 2.56-2.99 | 1.5-1.75 or 3-3.25 | <1.5 or >3.25 |
| P(mmol/L) | 0.85-1.51 | <0.85 or>1.51 | <0.5 or >1.75 | <0.3 or >3 |
| Mg(mmol/L) | 0.75-1.02 | <0.75 or >1.02 | <0.5 or >3 | <0.4 or >5 |
| Procalcitonin | 0-0.1 | 0.11-0.2 | >0.2 | >0.5 |
| Hemoglobin (g/L) | male: 120-160; female: 110-150 | male：91-119; female：91-109 or male：161-164; female：151-159 | 50-90/male：165-184; female：160-164 | <50 or male：>185，female：>165 |
| HCT(%) | 40-50 | 20-40 or 50-60 | 15-20 or 60-65 | <15 or >65 |
| MCV (fl) | 82-100 |  | <82 or >100 |  |
| MCH(pg) | 27.0-34.0 |  | <27.0 or >34.0 |  |
| RDW (%) | 10.0-15.0 |  | <10 or >15 |  |
| WBC (×10^9/L) | 3.5-9.5 | 9.51-20 or 1.5-3.51 | >20 or 1-1.49 | <1 or >30 |
| Neutrophil count (×10^9/L) | 1.8-6.3 | <1.8 or >6.3 | <1.5 or >8 | <0.5 or >8.5 |
| Neutrophil ratio (%) | 40-75 | >75 or <40 | <37.5 or >80 | <12.5 or >85 |
| Lymphocyte count (×10^9/L) | 1.1-3.2 | >1 xULN or <1 xLLN | >6 | >7.5 |
| Lymphocyte ratio (%) | 20-50/normal | >1 xULN or <1 xLLN | >60 | >75 |
| Eosinophil count (×10^9/L) | 0.02-0.52 | ＞0.52 or＜0.02 | >1.5 | >5 |
| Eosinophil ratio (%) | 0.4-8 | >1 xULN or <1 xLLN | >15 | >50 |
| Basophil count (×10^9/L) | 0-0.06 | >1 xULN or <1 xLLN | >0.2 | >2 |
| Basophil ratio (%) | 0-1 | >1 xULN or <1 xLLN | >2 | >20 |
| Monocyte number (×10^9/L) | 0.1-0.6 | >1 xULN or <1 xLLN | >1 | >10 |
| Monocyte ratio (%) | 3.0-10.0 | >1 xULN or <1 xLLN | >20 | >80 |
| Platelet (×10^9/L) | 125-350 | 50-126 or 351-449 | 20-49 or 450-799 | ≥800 or <20 |
| MPV(fl) | 6.5-12 |  | beyond normal limits |  |
| PCT(ml/L) | 0.11-0.28 |  | beyond normal limits |  |
| PDW (fl) | 12.0-18.0 |  | beyond normal limits |  |
| CRP(mg/L) | ≤10 | 10--30 | 30-90 | >90 |
| BNP (pg/ml) | 0-100 | 100-200 | 200-1000 | >1000 |
| CK-MB (ng/ml) | 0-3.7 | >3.7 | >6 | >10 |
| Myohemoglobin (ng/ml) | 11.6-73 | >73 | >80 | >100 |
| Troponin I (ng/ml) | 0-0.06 | >0.06 | >0.2 | >0.4 |
